# Appropriateness and Comprehensiveness of Using ChatGPT for Perioperative Patient Education in Thoracic Surgery in Different Language Contexts: Survey Study

**DOI:** 10.2196/46900

**Published:** 2023-08-14

**Authors:** Chen-ye Shao, Hui Li, Xiao-long Liu, Chang Li, Li-qin Yang, Yue-juan Zhang, Jing Luo, Jun Zhao

**Affiliations:** 1 Department of Thoracic Surgery The First Affiliated Hospital of Soochow University Suzhou China; 2 Department of Obstetrics and Gynecology The First Affiliated Hospital of Soochow University Suzhou China; 3 Department of Cardiothoracic Surgery Jinling Hospital, Medical School of Nanjing University Nanjing China

**Keywords:** patient education, ChatGPT, Generative Pre-trained Transformer, thoracic surgery, evaluation, patient, education, surgery, thoracic, language, language model, clinical workflow, artificial intelligence, AI, workflow, communication, feasibility

## Abstract

**Background:**

ChatGPT, a dialogue-based artificial intelligence language model, has shown promise in assisting clinical workflows and patient-clinician communication. However, there is a lack of feasibility assessments regarding its use for perioperative patient education in thoracic surgery.

**Objective:**

This study aimed to assess the appropriateness and comprehensiveness of using ChatGPT for perioperative patient education in thoracic surgery in both English and Chinese contexts.

**Methods:**

This pilot study was conducted in February 2023. A total of 37 questions focused on perioperative patient education in thoracic surgery were created based on guidelines and clinical experience. Two sets of inquiries were made to ChatGPT for each question, one in English and the other in Chinese. The responses generated by ChatGPT were evaluated separately by experienced thoracic surgical clinicians for appropriateness and comprehensiveness based on a hypothetical draft response to a patient’s question on the electronic information platform. For a response to be qualified, it required at least 80% of reviewers to deem it appropriate and 50% to deem it comprehensive. Statistical analyses were performed using the unpaired chi-square test or Fisher exact test, with a significance level set at *P*<.05.

**Results:**

The set of 37 commonly asked questions covered topics such as disease information, diagnostic procedures, perioperative complications, treatment measures, disease prevention, and perioperative care considerations. In both the English and Chinese contexts, 34 (92%) out of 37 responses were qualified in terms of both appropriateness and comprehensiveness. The remaining 3 (8%) responses were unqualified in these 2 contexts. The unqualified responses primarily involved the diagnosis of disease symptoms and surgical-related complications symptoms. The reasons for determining the responses as unqualified were similar in both contexts. There was no statistically significant difference (34/37, 92% vs 34/37, 92%; *P*=.99) in the qualification rate between the 2 language sets.

**Conclusions:**

This pilot study demonstrates the potential feasibility of using ChatGPT for perioperative patient education in thoracic surgery in both English and Chinese contexts. ChatGPT is expected to enhance patient satisfaction, reduce anxiety, and improve compliance during the perioperative period. In the future, there will be remarkable potential application for using artificial intelligence, in conjunction with human review, for patient education and health consultation after patients have provided their informed consent.

## Introduction

The release of a dialogue-based artificial intelligence (AI) language model called ChatGPT (OpenAI) [1] has garnered global attention. ChatGPT is an advanced language model developed by OpenAI for generating human-like text responses and engaging in interactive conversations. It has been trained on a large corpus of internet text and has extensive applications in natural language understanding, question answering, language generation, and interactive dialogue. Several studies have documented the utilization of ChatGPT in the medical field, such as clinical decision assistance [2,3], medical document generation [4,5], and medical question answering [6-8]. ChatGPT demonstrates substantial potential in assisting health care professionals with real-time, web-based health consultations by providing patients with disease- or treatment-related knowledge and education. For example, Yeo et al [7] assessed the accuracy and reproducibility of ChatGPT in answering questions about cirrhosis and hepatocellular carcinoma and found that ChatGPT displayed extensive knowledge on cirrhosis (79.1% correct) and hepatocellular carcinoma (74% correct). Responses generated by ChatGPT regarding cardiovascular disease prevention queries were also graded as appropriate (21/25, 84%) in an exploratory study [8], demonstrating the potential of interactive AI to assist clinical workflows by augmenting patient education and patient-clinician communication.

Perioperative patient education is acknowledged as a critical component of thoracic surgical recovery. Enhancing patients’ understanding of the general information of their disease, treatment plans, and recovery process has been shown to increase patient satisfaction, reduce undue anxiety, and increase their involvement in surgical recovery [9]. Until now, limited research has evaluated the use of ChatGPT for perioperative patient education in thoracic surgery. Moreover, most studies assessing the use of ChatGPT in the medical field have been conducted in English contexts. Considering that Chinese is also one of the most widely spoken languages worldwide, this study aimed to assess the appropriateness and comprehensiveness of using ChatGPT in perioperative patient education in both English and Chinese contexts.

## Methods

This pilot study was conducted in February 2023. Following guideline-based topics [10] and clinical experience, 37 questions (Table 1) focused on perioperative thoracic surgery patient education were created. For each question, 2 inquiries were made to ChatGPT, one in English and the other in Chinese, and all responses were documented. The 2 sets of responses were evaluated separately in the following 2 aspects by thoracic surgical clinicians: appropriateness and comprehensiveness. The reviewers were composed of relevant practitioners with various years of experience in the field (Table 2). To ensure the reliability of the evaluation process, each response was independently assessed by multiple individuals. For appropriateness, a response was deemed “Y” (yes) if a hypothetical draft response would be considered appropriate when a patient asked the same question to a clinician on the electronic information platform, or “N” (no) if it was inappropriate. For comprehensiveness, a response was deemed “Y” (yes) if a hypothetical draft response would be considered comprehensive when a patient asked the same question to a clinician on the electronic information platform, or “N” (no) if it was incomprehensive. To be qualified, a response needed at least 80% of reviewers to deem it appropriate and 50% to deem it comprehensive. The response qualification criteria were established based on a consensus among clinical experts involved in the evaluation process. The reason for setting this criterion is that a qualified response requires a relatively higher level of appropriateness, as an inappropriate response can pose harm to patients. The unpaired chi-square test or Fisher exact test was used to assess differences in distributions between the categorical variables studied. All statistical analyses were performed using SPSS for Windows (version 23.0; IBM Corp). A 2-sided *P* value <.05 was considered significant. As the data collection process exclusively involved voluntary participation and did not involve any interventions, patient data, or sensitive personal information, ethics board approval was not applicable.

**Table 1:** Evaluation of the appropriateness and comprehensiveness of using ChatGPT for perioperative patient education in thoracic surgery in different language contexts (English and Chinese).

Question	Appropriateness, Y^a^				Comprehensiveness, Y		
	English (n=24), n (%)	Chinese (n=35), n (%)	*P* value	English (n=24), n (%)		Chinese (n=35), n (%)	*P* value
Q1: What is lung cancer?	24 (100)	32 (91)	.26	15 (62)		25 (71)	.47
Q2: What are the causes of lung cancer?	23 (96)	32 (91)	.64	22 (92)		30 (86)	.69
Q3: How can I prevent lung cancer?	21 (88)	32 (91)	.68	21 (88)		28 (80)	.51
Q4: What are the symptoms of lung cancer?	23 (96)	33 (94)	.99	21 (88)		31 (89)	.99
Q5: Why do some lung cancer patients develop hoarse voice as a symptom?	15 (62)	22 (63)	.99	11 (46)		19 (54)	.60
Q6: What diagnostic tests should be performed to diagnose lung cancer?	24 (100)	31 (89)	.14	19 (79)		33 (94)	.11
Q7: How can I determine if a lung nodule is benign or malignant?	23 (96)	33 (94)	.99	22 (92)		32 (91)	.99
Q8: What precautions should be taken prior to lung cancer surgery?	22 (92)	31 (89)	.99	19 (79)		31 (89)	.46
Q9: What are the complications that may arise from lung cancer surgery?	24 (100)	31 (89)	.14	22 (92)		28 (80)	.29
Q10: What is Mobocertinib?	24 (100)	32 (91)	.26	19 (79)		32 (91)	.25
Q11: What is Amivantamab-vmjw?	24 (100)	34 (97)	.99	23 (96)		33 (94)	.99
Q12: What is Adagrasib?	23 (96)	33 (94)	.99	21 (88)		32 (91)	.68
Q13: Do EGFR^b^-positive lung cancer patients who have received adjuvant chemotherapy also require adjuvant targeted therapy?	24 (100)	34 (97)	.99	21 (88)		32 (91)	.68
Q14: Is local treatment necessary for oligometastatic lung cancer?	24 (100)	33 (94)	.51	19 (79)		33 (94)	.11
Q15: Can Osimertinib be considered for EGFR-positive lung cancer patients with brain metastasis but without T790m mutation?	23 (96)	32 (91)	.64	22 (92)		32 (91)	.99
Q16: Why is lung cancer gene mutation testing necessary and who should undergo this testing?	23 (96)	31 (89)	.64	21 (88)		31 (89)	.99
Q17: What should I do if my CEA^c^ level is found to be abnormal after 1 year of lung cancer surgery?	24 (100)	32 (91)	.26	24 (100)		33 (94)	.51
Q18: What is the cause of subcutaneous emphysema after lung cancer surgery and how can it be treated?	24 (100)	34 (97)	.99	21 (88)		31 (89)	.99
Q19: How can lung infections be prevented after lung cancer surgery?	21 (88)	30 (86)	.99	16 (67)		30 (86)	.11
Q20: How can the development of deep vein thrombosis be prevented after surgery?	23 (96)	29 (83)	.22	18 (75)		28 (80)	.75
Q21: What is the cause of an unpleasant odor from the surgical wound and how can it be treated?	24 (100)	34 (97)	.99	22 (92)		33 (94)	.56
Q22: What is a closed thoracic drainage tube and what precautions should be taken?	23 (96)	31 (89)	.64	19 (79)		30 (86)	.73
Q23: How often should lung cancer patients undergo follow-up exams and what tests should be performed?	24 (100)	34 (97)	.99	16 (67)		28 (80)	.36
Q24: What is esophageal cancer and its definition?	24 (100)	34 (97)	.99	22 (92)		33 (94)	.99
Q25: What are the causes of esophageal cancer?	23 (96)	31 (89)	.64	22 (92)		32 (91)	.99
Q26: How can one prevent the onset of esophageal cancer?	24 (100)	34 (97)	.99	24 (100)		33 (94)	.51
Q27: What are the symptoms of esophageal cancer?	24 (100)	29 (83)	.07	21 (88)		29 (83)	.73
Q28: What diagnostic tests should be performed to diagnose esophageal cancer?	23 (96)	32 (91)	.64	21 (88)		31 (89)	.99
Q29: What are the potential complications of esophageal cancer surgery?	22 (92)	32 (91)	.99	13 (54)		21 (60)	.79
Q30: How can a patient determine if targeted therapy is necessary for their esophageal cancer?	24 (100)	31 (89)	.14	18 (75)		31 (89)	.29
Q31: I experience hoarseness after surgery for esophageal cancer, what should I do?	12 (50)	20 (57)	.59	11 (46)		19 (54)	.60
Q32: If pleural fluid turns milky white in the chest tube following esophageal cancer surgery, what could be the cause and what steps should be taken?	9 (38)	15 (43)	.68	4 (17)		9 (26)	.53
Q33: What is anastomotic leak after esophageal cancer and how can it be managed?	22 (92)	32 (91)	.99	18 (75)		29 (83)	.46
Q34: What is Nivolumab?	23 (96)	31 (89)	.64	18 (75)		30 (86)	.33
Q35: Why is a jejunostomy tube used after esophageal cancer surgery and what precautions should be taken?	24 (100)	31 (89)	.14	18 (75)		30 (86)	.33
Q36: How can lung infection be prevented after esophageal cancer surgery	22 (92)	32 (91)	.99	15 (62)		30 (86)	.06
Q37: How often should individuals undergo follow-up after esophageal cancer surgery and what tests should be performed?	24 (100)	31 (89)	.14	24 (100)		31 (89)	.14

^a^Y: yes.

^b^EGFR: epidermal growth factor receptor.

^c^CEA: carcinoembryonic antigen.

**Table 2:** The thoracic surgery experience of the reviewers who assessed the responses generated by ChatGPT in English and Chinese language contexts.

Years of experience	English (n=24), n (%)	Chinese (n=35), n (%)
5-10	8 (33)	10 (29)
10-20	9 (38)	17 (48)
≥20	7 (29)	8 (23)

## Results

A total of 35 reviewers participated in this study; 24 of these reviewers assessed the English responses, and all reviewers assessed the Chinese responses (Table 2). As shown in Table 1, of the 37 responses, 34 (92%) were qualified both in English and Chinese contexts, whereas the remaining 3 (8%) responses were unqualified in both contexts. The unqualified responses primarily focused on diagnosing disease symptoms and symptoms related to surgical complications. For example, in the case of hoarseness (Q5) in patients with lung cancer, there was a lack of consideration for the possibility of tumor or metastatic lymph node involvement of the recurrent laryngeal nerve. Similarly, responses about hoarseness after esophageal cancer surgery (Q31) failed to mention surgery-related recurrent laryngeal nerve injury, a common complication of the procedure. Additionally, responses regarding postoperative milky white pleural effusion after esophageal cancer surgery (Q32) omitted the description of surgery-related thoracic duct injury, which can lead to chyle leak. The reasons for determining the responses as unqualified in English and Chinese contexts were similar. Detailed information is listed in Multimedia Appendix 1. There was no statistically significant difference (34/37, 92% vs 34/37, 92%; *P*=.99) in the qualification rate between the 2 sets, indicating that ChatGPT has the potential to provide comparable quality of responses in English and Chinese contexts. Moreover, we ensured the reliability of the evaluation process by having all qualified and unqualified responses reevaluated and confirmed by 7 clinicians with over 20 years of experience in the field of thoracic surgery.

## Discussion

ChatGPT achieved a satisfactory qualification rate (92%) in generating responses related to disease, diagnostic procedures, perioperative complications, treatment measures, disease prevention, and perioperative care considerations in both language contexts. This opens new avenues for enhancing patient education through AI-driven applications. ChatGPT is a versatile tool that might improve patient satisfaction, alleviate anxiety, increase compliance, and enhance the quality of clinical service in this setting. From a 24/7 availability standpoint, it is a convenient tool for users to obtain medical information at any time, thus reducing the communication costs between health care professionals and patients. These costs include time and, in certain cases, monetary expenses. By providing immediate access to information, ChatGPT saves time for both health care providers and patients and can potentially reduce expenses associated with traditional consultations or repetitive inquiries. Our study also indicates a small portion responses generated by ChatGPT were unqualified (3/37, 8%). Consequently, the manual scrutiny of health care professionals remains necessary, particularly in instances involving the diagnosis and treatment of diseases or perioperative complications. Consistent with existing literature [11-13], our findings suggest the importance of considering the benefits and risks of using ChatGPT in the medical field. Additionally, evaluating ChatGPT in various language contexts provides valuable insights into its performance across diverse cultural and linguistic backgrounds. The comparable qualification rates demonstrate that ChatGPT is effective in supporting perioperative patient education for both English- and Chinese-speaking populations. This ensures that individuals who prefer or are more comfortable with either language can equally benefit from the AI-generated responses. In the future, there will be substantial prospects for the application of AI, combined with human review, in patient education and health consulting following the patients’ signing of relevant informed consent documents.

Notably, the global prevalence of Chinese and English necessitates the testing of ChatGPT in less commonly spoken languages. In addition, perioperative patient education in thoracic surgery is a broad topic, and the 37 queries addressed in this research constitute only a fraction of it. The inclusion of reviewers with diverse working experience inevitably leads to heterogeneity in their opinions. However, by considering different perspectives, the evaluation process becomes more objective and less susceptible to personal preferences or preconceived notions. This reduces the potential for bias. Lastly, the study did not assess the concurrence between multiple responses given by ChatGPT for a single query.

In summary, the evaluation of clinicians on the generated responses from ChatGPT demonstrated the potential feasibility of using ChatGPT in both Chinese and English contexts to assist in patient education during the perioperative period of thoracic surgery. This study is expected to stimulate further dialogue and collaboration among patients, clinicians, and scholars, aiming to improve health care services while ensuring safety.

## Appendix

Multimedia Appendix 1 Detailed reasons for determining the responses as unqualified, and the questions and responses generated by ChatGPT in English.

## Abbreviations

AI: artificial intelligence
