# Supplementary material for: Appropriateness and Comprehensiveness of Using ChatGPT for Perioperative Patient Education in Thoracic Surgery in Different Language Contexts: Survey Study
Source: Interact J Med Res. 2023 Aug 14;12:e46900. doi: 10.2196/46900 (PMC10463083; doi:10.2196/46900)
Supplement: Multimedia Appendix 1 [file ijmr_v12i1e46900_app1.pdf]

Supplementary material

The reasons for determining a response as unqualified include but are not limited to the following points:

Responses concerning hoarseness (Q5) in lung cancer patients did not take into account the possibility of tumor or metastatic lymph node involvement of the recurrent laryngeal nerve.

Responses regarding hoarseness after esophageal cancer surgery (Q31) lacked the description of surgery-related recurrent laryngeal nerve injury, which is a common complication of the procedure.

The responses regarding the postoperative milky white pleural effusion after esophageal cancer surgery (Q32) lacked the description of surgery-related thoracic duct injury, which can cause chyle leak, which is a common complication of the procedure.

Questions and the responses generated by chatGPT (only responses in English context were listed)

1. What is lung cancer?

LW 1. What is lung cancer?

Lung cancer is a type of cancer that begins in the lungs. It can either be a small cell lung cancer (SCLC), which is a fast-growing cancer, or a non-small cell lung cancer (NSCLC), which is a slower-growing cancer but more common. Lung cancer is caused by the abnormal growth of cells in the lungs, which can form tumors and interfere with the normal functioning of the lungs. Symptoms of lung cancer can include a persistent cough, chest pain, shortness of breath, and coughing up blood. The exact cause of lung cancer is not clear, but factors such as smoking, exposure to radon and air pollution, and a family history of lung cancer can increase the risk of developing the disease.

2. What are the causes of lung cancer?

LW What are the causes of lung cancer?

Lung cancer is caused by the uncontrolled growth of abnormal cells in the lungs. There are several factors that can increase the risk of developing lung cancer, including:

1. Tobacco smoking: Smoking is the leading cause of lung cancer and is responsible for about 85% of all cases.
2. Exposure to air pollution: Long-term exposure to air pollution, such as that from traffic, factories, and power plants, can increase the risk of lung cancer.
3. Exposure to radon: Radon is a naturally occurring radioactive gas that can seep into homes and increase the risk of lung cancer.
4. Family history: People with a family history of lung cancer are at a higher risk of developing the disease.
5. Occupational exposure: People who work in certain industries, such as mining, construction, and agriculture, may be exposed to chemicals that can increase the risk of lung cancer.
6. Personal history of lung disease: People with a history of lung disease, such as chronic obstructive pulmonary disease (COPD) or tuberculosis, are at a higher risk of developing lung cancer.
7. Previous cancer treatment: People who have been treated with radiation therapy for another type of cancer are at an increased risk of developing lung cancer.

It's important to note that not everyone who has risk factors for lung cancer will develop the disease, and many people with lung cancer have no known risk factors.

3. How can I prevent lung cancer?

LW How can I prevent lung cancer?

There are several steps you can take to reduce your risk of developing lung cancer:

1. Don't smoke: The single most effective way to prevent lung cancer is to not start smoking or to quit smoking if you already do.
2. Limit exposure to air pollution: Try to avoid outdoor activities in areas with high levels of air pollution, and make sure your home and workplace are well-ventilated.
3. Test your home for radon: Have your home tested for radon, and if high levels are found, have them reduced.
4. Eat a healthy diet: A diet rich in fruits, vegetables, and whole grains has been shown to lower the risk of lung cancer.
5. Exercise regularly: Regular physical activity has been linked to a lower risk of lung cancer.
6. Avoid exposure to carcinogens: If you work in an industry that exposes you to potentially cancer-causing chemicals, take precautions to minimize your exposure.
7. Get regular check-ups: If you are at higher risk for lung cancer due to smoking, exposure to carcinogens, or other risk factors, talk to your doctor about getting regular check-ups and screenings.

Remember that the best way to prevent lung cancer is to not smoke, and if you do smoke, to quit as soon as possible.

4. What are the symptoms of lung cancer?

LW What are the symptoms of lung cancer?

The symptoms of lung cancer can vary depending on the type and stage of the disease, but some common symptoms include:

1. Persistent cough: A cough that does not go away or gets worse over time can be a symptom of lung cancer.
2. Chest pain: Pain in the chest that worsens with deep breathing, coughing, or laughing can be a sign of lung cancer.
3. Shortness of breath: Feeling short of breath or experiencing a wheezing sound when breathing can be a symptom of lung cancer.
4. Coughing up blood: If you cough up blood, even just a small amount, it can be a sign of lung cancer.
5. Fatigue: Chronic fatigue and weakness can be symptoms of lung cancer.
6. Loss of appetite and weight loss: A loss of appetite and unexplained weight loss can be a sign of lung cancer.
7. Hoarseness: A change in your voice, such as hoarseness, can be a symptom of lung cancer.

It's important to note that these symptoms can also be caused by other conditions, so if you are experiencing any of these symptoms, it's important to see a doctor for a proper evaluation. Early detection of lung cancer is crucial for successful treatment and recovery.

5. Why do some lung cancer patients develop hoarse voice as a symptom?

LW Why do some lung cancer patients develop hoarse voice as a symptom?

Hoarseness can develop as a symptom of lung cancer because the tumors in the lungs can interfere with the normal functioning of the larynx, which is the voice box. The larynx contains the vocal cords, which produce sound when air passes over them. If a tumor in the lungs presses against the larynx, it can cause the vocal cords to become inflamed or paralyzed, leading to hoarseness.

In some cases, lung cancer can also spread to the larynx, causing hoarseness as a result. Additionally, treatment for lung cancer, such as radiation therapy, can also cause inflammation and swelling in the larynx, leading to hoarseness.

It's important to remember that hoarseness can also be caused by other conditions, such as laryngitis or an infection, so it's important to see a doctor for a proper evaluation if you are experiencing this symptom.

6. What diagnostic tests should be performed to diagnose lung cancer?

LW What diagnostic tests should be performed to diagnose lung cancer?

There are several diagnostic tests that can be performed to diagnose lung cancer, including:

1. Chest X-ray: A chest X-ray can show an abnormal mass in the lungs and can help determine the size and location of the tumor.
2. Computed Tomography (CT) scan: A CT scan is a more detailed imaging test that can provide a clearer picture of the lungs and can help determine the size and location of the tumor.
3. Magnetic Resonance Imaging (MRI) scan: An MRI scan uses magnetic fields and radio waves to produce detailed images of the body and can provide additional information about the tumor and surrounding tissues.
4. Positron Emission Tomography (PET) scan: A PET scan uses a small amount of radioactive material to produce images of the body and can help determine if the tumor is cancerous and if it has spread to other parts of the body.
5. Bronchoscopy: During a bronchoscopy, a flexible scope is inserted through the mouth or nose and into the lungs to examine the airways and obtain tissue samples for biopsy.
6. Biopsy: A biopsy involves removing a small piece of tissue from the lung for examination under a microscope. This is the most definitive way to diagnose lung cancer.

In some cases, a combination of these tests may be performed to diagnose lung cancer and determine the best course of treatment. The specific tests performed will depend on the individual patient and the suspected stage and type of lung cancer.

7. How can I determine if a lung nodule is benign or malignant?

LW

How can I determine if a lung nodule is benign or malignant?

A lung nodule is a small growth or lump that can be seen on an imaging test, such as a chest X-ray or a CT scan. The presence of a lung nodule does not necessarily indicate that a person has lung cancer, as many lung nodules are benign (non-cancerous). However, some lung nodules can be malignant (cancerous), so it's important to determine the nature of the nodule in order to properly diagnose and treat lung cancer.

To determine if a lung nodule is benign or malignant, a healthcare provider may use a combination of the following methods:

1. Observation: If the nodule is small and does not appear to be growing, a healthcare provider may simply observe the nodule over time to see if it changes.
2. Imaging tests: Additional imaging tests, such as a CT scan, may be performed to determine the size and shape of the nodule and to see if it has changed over time.
3. Biopsy: A biopsy, in which a small sample of tissue is removed from the nodule for examination under a microscope, is the most definitive way to determine if a lung nodule is benign or malignant.
4. Blood tests: Blood tests can be used to look for markers associated with lung cancer, such as elevated levels of certain substances produced by cancer cells.

It's important to note that not all lung nodules need to be biopsied, and the specific course of action will depend on the individual patient and the suspected stage and type of lung cancer. A healthcare provider can help determine the best approach for diagnosing a lung nodule and treating lung cancer.

8. What precautions should be taken prior to lung cancer surgery?

LW What precautions should be taken prior to lung cancer surgery?

Lung cancer surgery, also known as thoracic surgery, is a major procedure that requires careful preparation. Some of the precautions that should be taken prior to lung cancer surgery include:

1. Quitting smoking: If you are a smoker, it's important to quit smoking prior to surgery, as smoking can slow down the healing process and increase the risk of complications.
2. Medications: Your healthcare provider will provide specific instructions regarding the use of any medications you are taking, including over-the-counter medications and supplements. Some medications may need to be discontinued or adjusted prior to surgery.
3. Vaccinations: You may need to receive certain vaccinations, such as the flu shot or the pneumococcal vaccine, prior to surgery to reduce the risk of infection.
4. Tests: You may need to undergo a variety of tests, such as blood tests, a chest X-ray, or a CT scan, prior to surgery to determine your overall health and to identify any potential complications.
5. Diet: Your healthcare provider may advise you to follow a specific diet prior to surgery to ensure that your body is adequately nourished and prepared for the procedure.
6. Preoperative instructions: Your healthcare provider will provide specific instructions regarding what you should and should not do prior to surgery, including information about fasting, drinking fluids, and taking medications.

It's important to follow all of the instructions provided by your healthcare provider to ensure a safe and successful outcome. If you have any questions or concerns, be sure to discuss them with your healthcare provider prior to your surgery.

9. What are the complications that may arise from lung cancer surgery?

LW What are the complications that may arise from lung cancer surgery?

Lung cancer surgery, also known as thoracic surgery, is a major procedure that carries certain risks and potential complications. Some of the most common complications that may arise from lung cancer surgery include:

1. Pain: Pain in the chest and shoulder is a common side effect of lung cancer surgery, but it can usually be managed with pain medications.
2. Infection: As with any surgical procedure, there is a risk of infection, which can be reduced by following proper hygiene and wound care instructions.
3. Bleeding: Bleeding during or after surgery is a potential complication, and in some cases, a blood transfusion may be necessary.
4. Blood clots: Blood clots can form in the legs or lungs, and may require anticoagulant medications or other treatments to prevent or manage.
5. Air leaks: The lungs may leak air following surgery, which can cause difficulty breathing and may require additional procedures to correct.
6. Pneumonia: Pneumonia, an infection in the lungs, is a potential complication following lung cancer surgery.
7. Nerve damage: Surgery can cause damage to the nerves in the chest, which may result in numbness, tingling, or other sensations in the chest or arms.
8. Pulmonary complications: Complications such as atelectasis (collapsed lung) and pneumonia may occur, particularly if the patient has reduced lung function prior to surgery.

It's important to note that not all patients will experience these complications, and the specific risks will depend on the individual patient and the extent of the surgery. Your healthcare provider can provide more information about the specific risks and potential complications of lung cancer surgery, and can help you prepare for and manage any side effects that may occur.

10. What is Mobocertinib?

LW What is Mobocertinib?

Mobocertinib is a type of medication used to treat non-small cell lung cancer (NSCLC). It is a tyrosine kinase inhibitor, which means that it works by blocking the activity of certain enzymes involved in cancer cell growth and division.

Mobocertinib is approved for the treatment of advanced or metastatic NSCLC with specific genetic mutations, including patients with epidermal growth factor receptor (EGFR) exon 20 insertion mutations. This medication is taken orally and is used in combination with other treatments, such as chemotherapy or radiation therapy, to improve outcomes for patients with NSCLC.

It's important to note that Mobocertinib is only recommended for use in patients with specific genetic mutations, and its use should be guided by a healthcare professional who is experienced in the treatment of lung cancer. Additionally, Mobocertinib can cause side effects, and it is important to discuss any potential risks and benefits of this medication with your healthcare provider prior to starting treatment.

11. What is Amivantamab-vmjw?

LW What is Amivantamab-vmjw

Amivantamab-vmjw is a type of medication used in the treatment of non-small cell lung cancer (NSCLC). It is a monoclonal antibody, which means that it is a type of protein that is designed to target specific cells in the body.

Amivantamab-vmjw is used to treat patients with advanced or metastatic NSCLC who have a specific genetic mutation known as epidermal growth factor receptor (EGFR) exon 20 insertion mutations. It works by blocking the activity of EGFR, a protein that is involved in the growth and division of cancer cells, which can slow down or stop the progression of the disease.

Amivantamab-vmjw is given intravenously and may be used in combination with other treatments, such as chemotherapy, to improve outcomes for patients with NSCLC. However, it is important to note that this medication is only approved for use in patients with specific genetic mutations, and its use should be guided by a healthcare professional who is experienced in the treatment of lung cancer.

Like all medications, Amivantamab-vmjw can cause side effects, and it is important to discuss any potential risks and benefits of this medication with your healthcare provider prior to starting treatment.

12. What is Adagrasib?

LW What is Adagrasib?

Adagrasib is a type of medication used to treat non-small cell lung cancer (NSCLC). It is a selective inhibitor of an enzyme called GRK2 (G protein-coupled receptor kinase 2), which is involved in the regulation of certain proteins in the body.

Adagrasib is used to treat patients with advanced or metastatic NSCLC who have a specific genetic mutation known as KRAS G12C. KRAS is a gene that is involved in the growth and division of cells, and mutations in this gene are commonly found in many types of cancer, including NSCLC.

Adagrasib works by inhibiting GRK2, which allows for the activation of KRAS signaling, and thereby leads to the death of cancer cells. This medication is taken orally and may be used in combination with other treatments, such as chemotherapy or radiation therapy, to improve outcomes for patients with NSCLC.

It is important to note that Adagrasib is only approved for use in patients with KRAS G12C mutations, and its use should be guided by a healthcare professional who is experienced in the treatment of lung cancer. Additionally, Adagrasib can cause side effects, and it is important to discuss any potential risks and benefits of this medication with your healthcare provider prior to starting treatment.

13. Do EGFR-positive lung cancer patients who have received adjuvant chemotherapy also require adjuvant targeted therapy?

LW

Do EGFR-positive lung cancer patients who have received adjuvant chemotherapy also require adjuvant targeted therapy

The use of adjuvant targeted therapy in EGFR-positive lung cancer patients who have received adjuvant chemotherapy is a topic of ongoing research and debate among healthcare professionals.

Adjuvant chemotherapy is often used after surgery to treat early-stage non-small cell lung cancer (NSCLC) to reduce the risk of recurrence and improve overall survival. However, for patients with EGFR mutations, targeted therapy may also play an important role in improving outcomes.

Adjuvant targeted therapy with drugs such as gefitinib or erlotinib has been shown to be effective in prolonging progression-free survival and overall survival in patients with EGFR-positive NSCLC. However, the optimal duration of adjuvant targeted therapy is not yet well established, and the use of adjuvant targeted therapy in combination with adjuvant chemotherapy is still being studied.

The decision to use adjuvant targeted therapy in addition to adjuvant chemotherapy should be made in collaboration with a healthcare professional who is experienced in the treatment of lung cancer, taking into consideration the patient's individual circumstances, including the stage and specific genetic mutations of the cancer, and the patient's overall health and treatment goals.

It is important for patients with EGFR-positive NSCLC to discuss their treatment options with their healthcare team, including the potential benefits and risks of adjuvant targeted therapy, in order to make an informed decision about their care.

14. Is local treatment necessary for oligometastatic lung cancer?

LW

Is local treatment necessary for oligometastatic lung cancer

The role of local treatment for oligometastatic lung cancer is a topic of ongoing research and debate among healthcare professionals.

Oligometastatic lung cancer refers to a condition where a patient has limited numbers of metastases (spread of cancer from the primary site to other parts of the body), and the treatment options can vary depending on the specific circumstances of each case.

In some cases, local treatment, such as surgery or radiation therapy, may be necessary to control the primary tumour and prevent further spread of the cancer. This may be especially true if the patient has a single metastasis that can be treated with a local procedure, or if the patient is not a candidate for systemic treatments, such as chemotherapy or immunotherapy.

On the other hand, for some patients with oligometastatic lung cancer, systemic treatments, such as chemotherapy, immunotherapy, or targeted therapy, may be the preferred approach. The use of systemic treatments can be especially beneficial for patients with multiple metastases or those who are not eligible for local treatments due to medical or other reasons.

The choice of treatment for oligometastatic lung cancer should be made in collaboration with a healthcare professional who is experienced in the treatment of lung cancer, taking into consideration the patient's individual circumstances, including the stage and specific genetic mutations of the cancer, and the patient's overall health and treatment goals.

It is important for patients with oligometastatic lung cancer to discuss their treatment options with their healthcare team, including the potential benefits and risks of local and systemic treatments, in order to make an informed decision about their care.

15. Can Osimertinib be considered for EGFR-positive lung cancer patients with brain metastasis but without T790m mutation?

LW

Can Osimertinib be considered for EGFR-positive lung cancer patients with brain metastasis but without T790m mutation

The use of osimertinib in EGFR-positive lung cancer patients with brain metastasis but without T790M mutation is a topic of ongoing research and debate among healthcare professionals.

Osimertinib is a third-generation tyrosine kinase inhibitor (TKI) that specifically targets the EGFR T790M mutation, which is a common resistance mechanism to first- and second-generation EGFR TKIs. Therefore, it is typically used in patients with EGFR-positive non-small cell lung cancer (NSCLC) who have developed resistance to earlier TKI treatments and have been found to have the T790M mutation.

For patients with EGFR-positive NSCLC who have brain metastasis and do not have the T790M mutation, the use of osimertinib may not be appropriate, as it may not effectively target the cancer cells in this patient population.

In these cases, other treatment options, such as whole-brain radiation therapy, surgical resection, or systemic treatments, such as chemotherapy or immunotherapy, may be considered. The choice of treatment should be made in collaboration with a healthcare professional who is experienced in the treatment of lung cancer and brain metastasis, taking into consideration the patient's individual circumstances, including the stage and specific genetic mutations of the cancer, and the patient's overall health and treatment goals.

16. Why is lung cancer gene mutation testing necessary and who should undergo this testing?

LW

Why is lung cancer gene mutation testing necessary and who should undergo this testing

Lung cancer gene mutation testing is important because it can provide valuable information about the genetic makeup of a patient's cancer, which can help guide treatment decisions.

In some cases, specific genetic mutations, such as those in the EGFR gene, can predict how a patient will respond to certain treatments, such as targeted therapies. Knowing the specific genetic mutations present in a patient's cancer can also help identify potential new treatment options and inform clinical trial eligibility.

Not all patients with lung cancer will benefit from gene mutation testing, and the decision to undergo testing should be made in collaboration with a healthcare professional who is experienced in the treatment of lung cancer.

The following patient populations are typically considered for lung cancer gene mutation testing:

1. Patients with non-small cell lung cancer (NSCLC), which makes up the majority of lung cancers.
2. Patients with advanced or recurrent lung cancer, who may benefit from the identification of specific genetic mutations that can inform treatment decisions.
3. Patients who are candidates for targeted therapies, such as tyrosine kinase inhibitors (TKIs), which can be effective in treating certain genetic mutations.
4. Patients who are considering participating in clinical trials for new treatments, as many trials require patients to have specific genetic mutations.

It is important for patients to discuss their individual circumstances with their healthcare team, including their specific type and stage of lung cancer, to determine if gene mutation testing is appropriate for them.

17. What should I do if my CEA level is found to be abnormal after 1 year of lung cancer surgery?

LW

What should I do if my CEA level is found to be abnormal after 1 year of lung cancer surgery?

If your CEA (carcinoembryonic antigen) level is found to be abnormal after one year of lung cancer surgery, it is important to follow up with your healthcare provider as soon as possible.

CEA is a protein that is often elevated in individuals with certain types of cancer, including lung cancer. Elevated levels of CEA after lung cancer surgery may indicate that the cancer has returned (recurrence) or that a new cancer has developed.

Your healthcare provider may recommend further testing, such as imaging studies (e.g. CT scan, PET scan), biopsy, or other laboratory tests, to determine the cause of the elevated CEA level and to determine the best course of action.

Based on the results of these tests, your healthcare provider may recommend additional treatment, such as radiation therapy, chemotherapy, or another surgery. It is important to follow your healthcare provider's recommendations and to attend all recommended follow-up appointments to monitor your CEA levels and to detect any changes as early as possible.

It is also important to maintain a healthy lifestyle, such as engaging in regular physical activity, eating a well-balanced diet, and avoiding smoking and excessive alcohol consumption, to help support overall health and wellness.

If you have concerns or questions about your CEA levels, it is important to discuss them with your healthcare provider, who can provide you with more information and guidance.

18. What is the cause of subcutaneous emphysema after lung cancer surgery and how can it be treated?

LW

What is the cause of subcutaneous emphysema after lung cancer surgery and how can it be treated?

Subcutaneous emphysema is a condition that occurs when air or gas becomes trapped under the skin, typically as a result of injury or trauma to the airways. It can occur after lung cancer surgery, particularly after thoracic surgery.

The cause of subcutaneous emphysema after lung cancer surgery is usually a result of a tear in the airway or lung tissue, which allows air to escape into the surrounding tissue. This can happen during the surgical procedure or in the postoperative period.

Treatment for subcutaneous emphysema will depend on the severity of the condition and the underlying cause. In some cases, no treatment may be necessary, as the air will eventually be reabsorbed by the body. However, in more severe cases, treatment may include:

1. Observation: Monitoring the patient for any changes or worsening of symptoms.
2. Pain management: Pain medication can help to relieve any discomfort associated with subcutaneous emphysema.
3. Oxygen therapy: Providing supplemental oxygen can help to improve oxygenation and support the healing process.
4. Drainage: If a large amount of air has accumulated, it may be necessary to remove it through drainage.
5. Surgery: In severe cases, surgery may be necessary to repair any tears in the airways or lungs.

It is important to discuss any symptoms of subcutaneous emphysema with your healthcare provider, who can determine the best course of treatment for your individual needs.

19. How can lung infections be prevented after lung cancer surgery?

LW How can lung infections be prevented after lung cancer surgery?

Lung infections are a common complication after lung cancer surgery, and they can be serious and even life-threatening. However, there are steps that can be taken to help prevent lung infections after surgery:

1. Quit smoking: If you smoke, quitting before and after surgery can help reduce your risk of infection.
2. Get vaccinated: Certain vaccinations, such as the pneumococcal vaccine and the influenza vaccine, can help protect against lung infections.
3. Practice good hygiene: Wash your hands frequently, especially before and after touching your face or mouth, to help prevent the spread of germs.
4. Exercise: Regular exercise can help improve your overall lung function and help prevent infections.
5. Take antibiotics as prescribed: If you are prescribed antibiotics after surgery, it is important to take them as directed to help prevent infections.
6. Seek prompt treatment for any symptoms of infection: If you experience symptoms of a lung infection, such as fever, coughing, or shortness of breath, it is important to seek prompt medical treatment.

It is also important to maintain close follow-up with your healthcare provider after lung cancer surgery to monitor your progress and detect any potential infections early. By taking these steps, you can help reduce your risk of lung infections and promote a successful recovery after lung cancer surgery.

20. How can the development of deep vein thrombosis be prevented after surgery?

LW How can the development of deep vein thrombosis be prevented after surgery?

Deep vein thrombosis (DVT) is a serious condition that can occur after surgery and is caused by the formation of blood clots in the deep veins of the body, usually in the legs. To prevent the development of DVT after surgery, there are several steps that can be taken:

1. Keep moving: Encourage yourself to move around as much as possible after surgery, with the guidance of your healthcare provider. This can help improve circulation and prevent blood clots.
2. Wear compression stockings: Your healthcare provider may recommend wearing compression stockings after surgery to improve blood flow and prevent blood clots.
3. Take medications as prescribed: Anticoagulants, also known as blood thinners, may be prescribed after surgery to help prevent blood clots. It is important to take these medications exactly as prescribed by your healthcare provider.
4. Maintain a healthy weight: Being overweight or obese can increase your risk of DVT, so it is important to maintain a healthy weight.
5. Avoid extended periods of sitting or lying down: Try to avoid sitting or lying down for extended periods of time, especially on long flights or car trips.
6. Keep your legs elevated: Whenever possible, keep your legs elevated to help improve circulation.

It is also important to be aware of the symptoms of DVT, which can include swelling, warmth, redness, and pain in the affected leg. If you experience any of these symptoms, it is important to seek prompt medical attention. By taking these steps, you can help reduce your risk of developing DVT after surgery.

21. What is the cause of an unpleasant odor from the surgical wound and how can it be treated?

LW

What is the cause of an unpleasant odor from the surgical wound and how can it be treated?

An unpleasant odor from a surgical wound can be caused by a number of factors, including infection, wound dehiscence (when the wound opens up), or necrosis (death of tissue). Here are some steps that can be taken to treat an unpleasant odor from a surgical wound:

1. Keep the wound clean: Keeping the wound clean is the first step in preventing and treating an unpleasant odor. Gently wash the wound with soap and water, and change the dressing regularly.
2. Use an antiseptic: Your healthcare provider may recommend using an antiseptic solution, such as hydrogen peroxide or betadine, to help prevent infection and reduce the odor.
3. Avoid exposing the wound to moisture: Keep the wound dry and covered to prevent exposure to moisture, which can promote the growth of bacteria.
4. Use a topical antibiotic: If the wound appears infected, your healthcare provider may prescribe a topical antibiotic to help reduce the odor.
5. Manage pain: Pain or discomfort in the wound can make the odor worse, so it is important to manage pain effectively.
6. Seek prompt medical attention: If the odor is accompanied by other symptoms, such as increasing pain, redness, swelling, or fever, it is important to seek prompt medical attention. These symptoms may indicate an infection, which requires prompt treatment.

In some cases, an unpleasant odor from a surgical wound may resolve on its own. However, if the odor persists or worsens, it is important to seek medical attention to determine the underlying cause and to receive appropriate treatment.

22. What is a closed thoracic drainage tube and what precautions should be taken?

LW

What is a closed thoracic drainage tube and what precautions should be taken?

A closed thoracic drainage tube is a thin, flexible tube that is inserted into the chest after lung surgery to remove air, fluid, or blood that may have accumulated in the pleural cavity. The tube is connected to a collection chamber that sits outside the body and measures the amount of fluid or air being removed.

To prevent complications, it is important to follow the precautions recommended by your healthcare provider, which may include:

1. Keep the collection chamber at the level of the chest or slightly below: This helps prevent fluid or air from flowing back into the pleural cavity.
2. Avoid pulling or tugging on the tube: This can cause the tube to become dislodged or damaged, leading to complications.
3. Avoid twisting or kinking the tube: This can obstruct the flow of fluid or air and cause complications.
4. Keep the collection chamber clean and dry: This helps prevent infections.
5. Notify your healthcare provider immediately if the tube becomes dislodged or if there is any increase in pain or discomfort in the area around the tube.
6. Follow any additional instructions provided by your healthcare provider, such as how often to empty the collection chamber, how to change the dressing around the insertion site, and when to discontinue use of the drainage tube.

It is important to follow these precautions and to report any concerns or complications to your healthcare provider promptly to ensure the safe and effective use of a closed thoracic drainage tube after lung surgery.

23. How often should lung cancer patients undergo follow-up exams and what tests should be performed?

LW

How often should lung cancer patients undergo follow-up exams and what tests should be performed?

The frequency and types of follow-up exams for lung cancer patients will depend on several factors, including the stage of the cancer, the patient's overall health, and the type of treatment received.

Typically, lung cancer patients will undergo regular follow-up exams, including physical exams, imaging tests (such as chest x-rays or CT scans), and blood tests to monitor their health and check for any signs of disease recurrence. The frequency of these exams will be determined by the healthcare provider and may range from every few months to once a year or more.

In general, it is important for lung cancer patients to undergo regular follow-up exams to monitor their health and to detect any changes or signs of recurrence as early as possible. This allows for prompt and effective treatment, if necessary, and helps to improve the patient's overall outcomes.

It is also important for lung cancer patients to maintain regular communication with their healthcare provider and to report any symptoms or concerns as soon as they arise.

24. What is esophageal cancer and its definition?

LW

What is esophageal cancer and its definition?

Esophageal cancer is a type of cancer that starts in the cells lining the esophagus, which is the muscular tube that connects the mouth to the stomach. The esophagus is responsible for moving food and liquid from the mouth to the stomach for digestion.

Esophageal cancer can be classified into two main types, squamous cell carcinoma and adenocarcinoma, based on the type of cells in which the cancer originates. Squamous cell carcinoma starts in the flat cells that line the esophagus, while adenocarcinoma starts in the glandular cells that produce and release mucus and other fluids.

Esophageal cancer is a serious condition that can cause a range of symptoms, including difficulty swallowing (dysphagia), weight loss, chest pain, and persistent coughing or hoarseness. If left untreated, esophageal cancer can spread to other parts of the body and become life-threatening.

Diagnosis of esophageal cancer typically involves a combination of medical history, physical examination, imaging tests, and biopsy. Treatment options for esophageal cancer may include surgery, radiation therapy, chemotherapy, or a combination of these. The specific treatment approach will depend on the stage and location of the cancer, as well as the patient's overall health.

25. What are the causes of esophageal cancer?

LW What are the causes of esophageal cancer?

The exact cause of esophageal cancer is not well understood, but there are several known risk factors that increase the likelihood of developing the disease. Some of the most common causes of esophageal cancer include:

1. Tobacco use: Smoking cigarettes or using other tobacco products increases the risk of developing esophageal cancer.
2. Alcohol consumption: Heavy drinking can increase the risk of developing esophageal cancer, particularly squamous cell carcinoma.
3. Barrett's esophagus: This condition occurs when the normal cells in the esophagus are replaced by abnormal cells, and it increases the risk of developing adenocarcinoma.
4. Gastroesophageal reflux disease (GERD): Chronic acid reflux can cause changes to the cells in the esophagus, which can increase the risk of developing esophageal cancer.
5. Diet: A diet that is low in fruits and vegetables and high in processed foods and red meat has been linked to a higher risk of developing esophageal cancer.
6. Obesity: Being overweight or obese increases the risk of developing esophageal cancer, especially adenocarcinoma.
7. Age: The risk of developing esophageal cancer increases with age, with the majority of cases occurring in people over the age of 50.

It's important to note that having one or more of these risk factors does not guarantee that someone will develop esophageal cancer. On the other hand, some people with no known risk factors may still develop the disease.

26. How can one prevent the onset of esophageal cancer?

LW How can one prevent the onset of esophageal cancer?

There are several steps that you can take to help reduce your risk of developing esophageal cancer:

1. Quit smoking: If you smoke, quitting is the single most important step you can take to reduce your risk of esophageal cancer and many other health problems.
2. Limit alcohol consumption: If you drink alcohol, do so in moderation. It's recommended that women have no more than one drink per day and men have no more than two.
3. Maintain a healthy weight: Being overweight or obese increases the risk of esophageal cancer, especially adenocarcinoma. Eating a healthy diet and getting regular exercise can help you maintain a healthy weight.
4. Eat a healthy diet: A diet that is rich in fruits, vegetables, and whole grains and low in processed foods, red meat, and sugar can help reduce the risk of esophageal cancer.
5. Control acid reflux: If you have gastroesophageal reflux disease (GERD), take steps to control your symptoms and prevent damage to the esophagus. This may include changes to your diet and lifestyle, medication, or surgery.
6. Get regular check-ups: If you have Barrett's esophagus or a history of GERD, it's important to get regular check-ups to monitor for any changes in the cells of the esophagus.
7. Avoid harmful substances: Exposure to certain harmful substances, such as tobacco smoke, alcohol, and chemicals, can increase the risk of developing esophageal cancer. Avoiding exposure to these substances can help reduce your risk.

27. What are the symptoms of esophageal cancer?

LW

What are the symptoms of esophageal cancer?

The symptoms of esophageal cancer can be similar to those of other conditions, but some common symptoms include:

1. Difficulty swallowing (dysphagia): You may have the sensation that food is getting stuck in your throat or chest, or you may choke while eating.
2. Painful swallowing (odynophagia)
3. Weight loss: You may lose weight without trying, and feel full faster when eating.
4. Chest pain: You may feel a burning or pressure sensation in your chest, particularly behind the breastbone.
5. Heartburn or acid reflux: You may experience a worsening of symptoms if you already have gastroesophageal reflux disease (GERD).
6. Hoarseness or sore throat: You may have trouble speaking, or a persistent sore throat.
7. Coughing or wheezing: You may cough up blood, or have difficulty breathing.
8. Vomiting: You may vomit food or blood.

It's important to keep in mind that these symptoms can also be caused by other conditions, and not all people with esophageal cancer will have the same symptoms. If you are experiencing any of these symptoms, it's important to talk to your doctor to determine the cause.

28. What diagnostic tests should be performed to diagnose esophageal cancer?

LW

What diagnostic tests should be performed to diagnose esophageal cancer?

The following tests may be performed to diagnose esophageal cancer:

1. Endoscopy: A flexible, lighted tube (endoscope) is inserted down your throat to examine the inside of your esophagus. This is often the first test performed to diagnose esophageal cancer.
2. Biopsy: During an endoscopy, your doctor may take a small sample of tissue (biopsy) from the suspicious area. This sample will be sent to a laboratory to determine if cancer cells are present.
3. Barium swallow: You will drink a liquid that contains barium, which will coat the inside of your esophagus. X-rays will be taken to see if there are any abnormalities in the esophagus.
4. CT scan: A CT scan creates detailed cross-sectional images of your body. Your doctor may use this test to see if the cancer has spread to other parts of your body.
5. PET scan: A PET scan is a type of imaging test that uses a small amount of radioactive material to produce detailed images of your body. This test can help your doctor determine if the cancer has spread to other parts of your body.
6. Endoscopic ultrasound: An endoscopic ultrasound uses sound waves to create images of the inside of your esophagus. Your doctor may use this test to determine the depth of the cancer and whether it has spread to the surrounding tissues.

Your doctor will determine which tests are best for you based on your specific symptoms and medical history.

29. What are the potential complications of esophageal cancer surgery?

LW What are the potential complications of esophageal cancer surgery?

Esophageal cancer surgery can have several potential complications, including:

1. Leakage from the surgical site: This can occur if the connection between the remaining portion of the esophagus and the stomach does not heal properly.
2. Stricture formation: Scar tissue can form at the surgical site and cause the esophagus to narrow, making it difficult to swallow.
3. Chylothorax: This is a rare but serious complication in which lymphatic fluid accumulates in the chest cavity, causing difficulty breathing.
4. Pneumonia: Pneumonia is an infection in the lungs that can occur after surgery, especially in patients who are unable to fully clear their airways due to the presence of a breathing tube.
5. Anastomotic dehiscence: This is a rare but serious complication in which the connection between the remaining portion of the esophagus and the stomach tears open.
6. Wound infection: An infection can develop at the surgical site and cause discomfort and delay healing.
7. Heart or lung problems: The stress of surgery and anesthesia can cause heart or lung problems, especially in patients with pre-existing conditions.
8. Reaction to anesthesia: Some patients may have a reaction to the anesthesia used during surgery, causing symptoms such as nausea, vomiting, or breathing difficulties.

It's important to talk to your doctor about the potential risks and benefits of esophageal cancer surgery, and to carefully follow your doctor's instructions for preparation and aftercare to minimize the risk of complications.

30. How can a patient determine if targeted therapy is necessary for their esophageal cancer?

LW How can a patient determine if targeted therapy is necessary for their esophageal cancer?

The decision to use targeted therapy for esophageal cancer depends on several factors, including the type and stage of the cancer, as well as the patient's overall health and medical history. The presence of certain genetic mutations or biomarkers in the cancer cells can also influence the decision to use targeted therapy.

A patient with esophageal cancer may undergo genetic testing of the cancer cells to identify any mutations or biomarkers that can be targeted with specific drugs. The results of this testing can help the patient's doctor determine if targeted therapy is appropriate and what type of targeted therapy would be most effective.

It's important to work closely with your doctor to determine the best treatment plan for your specific situation, and to carefully consider the benefits and risks of targeted therapy and other treatments before making a decision.

31. I experience hoarseness after surgery for esophageal cancer, what should I do?

LW I experience hoarseness after surgery for esophageal cancer, what should I do?

Hoarseness after surgery for esophageal cancer may be a sign of injury to the larynx or voice box, which is located near the esophagus. If you experience hoarseness after your surgery, it's important to seek medical attention as soon as possible.

Your doctor may perform a laryngoscopy to examine your larynx and determine the cause of your hoarseness. Depending on the cause, treatment may involve rest, speech therapy, or additional medical or surgical interventions.

It's also important to maintain good communication with your doctor and follow their recommendations for postoperative care to minimize any complications and promote healing. If you have any concerns or questions about your symptoms, don't hesitate to reach out to your doctor.

32. If pleural fluid turns milky white in the chest tube following esophageal cancer surgery, what could be the cause and what steps should be taken?

LW

If pleural fluid turns milky white in the chest tube following esophageal cancer surgery, what could be the cause and what steps should be taken?

If pleural fluid in the chest tube following esophageal cancer surgery turns milky white, it could indicate that there is an infection or accumulation of pus in the pleural cavity. This is a serious complication and requires prompt medical attention. The patient should contact their surgeon or healthcare provider immediately. They may need to undergo further diagnostic tests, such as a pleural fluid culture, to determine the cause of the infection. They may also need to receive antibiotics or other treatments to clear the infection. In some cases, a chest tube may need to be reinserted to drain the fluid.

33. What is anastomotic leak after esophageal cancer and how can it be managed?

LW

What is anastomotic leak after esophageal cancer and how can it be managed?

An anastomotic leak is a complication that can occur after surgery for esophageal cancer. It refers to a break in the connection between the new connection created between the esophagus and stomach (anastomosis) after the removal of the cancerous portion of the esophagus. This can result in food, air, or digestive juices leaking from the anastomosis into the chest cavity.

Anastomotic leaks can cause symptoms such as fever, chest or abdominal pain, and difficulty swallowing. If left untreated, an anastomotic leak can lead to serious complications such as infection, abscess formation, and sepsis.

The management of an anastomotic leak typically involves close monitoring and management of symptoms, as well as antibiotics to prevent or treat infection. In some cases, a patient may require additional surgery to repair the leak. Treatment will depend on the severity and location of the leak, and will be determined by the patient's healthcare provider. It is important to discuss any symptoms or concerns with your healthcare provider in a timely manner so that appropriate treatment can be started as soon as possible.

34. What is Nivolumab?

LW What is Nivolumab?

Nivolumab is a type of immunotherapy drug that is used to treat cancer. It is a monoclonal antibody that works by blocking the activity of a protein called PD-1, which is found on the surface of certain immune cells called T cells. By blocking PD-1, Nivolumab allows the immune system to better recognize and attack cancer cells. Nivolumab has been approved for the treatment of several types of cancer, including melanoma, non-small cell lung cancer, renal cell carcinoma, classical Hodgkin lymphoma, and others. It is usually given as an intravenous infusion and is typically administered in combination with other cancer treatments, such as chemotherapy or radiation therapy.

35. Why is a jejunostomy tube used after esophageal cancer surgery and what precautions should be taken?

LW Why is a jejunostomy tube used after esophageal cancer surgery and what precautions should be taken?

A jejunostomy tube is used after esophageal cancer surgery as a form of feeding support in cases where the patient is unable to eat or swallow food normally. The tube is inserted through the abdominal wall into the jejunum, which is the part of the small intestine closest to the stomach. This allows food and liquids to bypass the esophagus and directly enter the small intestine for nutrition.

The following precautions should be taken when using a jejunostomy tube:

1. Proper placement: The placement of the jejunostomy tube should be confirmed through X-ray or other imaging studies to ensure it is in the correct location.
2. Care of the tube site: The tube site should be regularly checked for redness, swelling, or discharge, and any changes should be reported to the healthcare provider immediately.
3. Prevention of infection: Good hygiene practices and regular cleaning of the tube and surrounding area can help prevent infection.
4. Monitoring fluid and electrolyte levels: The patient's fluid and electrolyte levels should be monitored regularly, as the direct infusion of nutrients into the small intestine can lead to imbalances.
5. Checking for blockages: The flow of food and liquids through the jejunostomy tube should be checked regularly to ensure it is not blocked.

It is important to follow the instructions and guidelines provided by the healthcare team to ensure proper use of the jejunostomy tube and minimize any potential complications.

36. How can lung infection be prevented after esophageal cancer surgery?

LW

How can lung infection be prevented after esophageal cancer surgery?

There are several steps that can be taken to prevent lung infections after esophageal cancer surgery:

1. Maintaining good hygiene: Patients should regularly wash their hands to prevent the spread of bacteria.
2. Preventing aspiration: Eating and drinking slowly, avoiding lying down for at least 2 hours after eating, and avoiding foods and liquids that are more likely to cause aspiration can help prevent lung infections.
3. Encouraging deep breathing and coughing: Regular deep breathing and coughing can help keep the lungs clear of secretions and reduce the risk of infection.
4. Antibiotic prophylaxis: In some cases, patients may be prescribed antibiotics to prevent lung infections.
5. Avoiding smoking: Smoking can weaken the immune system and increase the risk of lung infections.
6. Monitoring for signs of infection: Patients should be aware of the symptoms of lung infections and seek medical attention promptly if they develop any symptoms.

It's important to follow the instructions of your healthcare provider and report any concerns or symptoms promptly to prevent and manage potential complications after esophageal cancer surgery.

37.How often should individuals undergo follow-up after esophageal cancer surgery and what tests should be performed?

LW

How often should individuals undergo follow-up after esophageal cancer surgery and what tests should be performed?

Following esophageal cancer surgery, regular follow-up appointments are important to monitor for any recurrence of the cancer or development of new complications. The frequency and type of follow-up exams may vary depending on the individual's specific case and treatment plan.

Typically, follow-up appointments may occur every 3-6 months in the first 2 years after surgery, and then once or twice a year thereafter. During these appointments, the following tests may be performed:

- Physical exam: To check for any changes in the patient's physical appearance and to assess the surgical site for any signs of recurrence.
- Endoscopy: An endoscope (a thin, flexible tube with a camera) may be used to look inside the patient's esophagus and stomach to check for any signs of recurrence or complications.
- Imaging tests: Imaging tests such as X-rays, CT scans, or PET scans may be performed to monitor for any changes in the patient's chest or abdomen and to check for any signs of recurrence or spread of the cancer.
- Blood tests: Blood tests may be performed to check for any changes in the patient's blood cell counts or liver and kidney function.

It's important to note that the frequency and type of follow-up exams may vary depending on the individual's specific case, so it's best to consult with the patient's healthcare team to determine the best follow-up plan.
